# Investigating Nutrition and Hydration Knowledge and Practice among a Cohort of Age-Grade Rugby Union Players

**DOI:** 10.3390/nu16040533

**Published:** 2024-02-14

**Authors:** Shane Scanlon, Catherine Norton

**Affiliations:** 1 Department of Physical Education and Sport Sciences, University of Limerick, V94 T9PX Limerick, Ireland; 2 Health Research Institute, University of Limerick, V94 T9PX Limerick, Ireland; 3 Sport & Human Performance Research Centre, University of Limerick, V94 T9PX Limerick, Ireland

**Keywords:** rugby union, nutrition, hydration, knowledge, practice, adolescent

## Abstract

Optimal athletic performance relies on meeting specific nutritional requirements, encompassing adequate calorie intake, macronutrient intake, and hydration. Misinformation or misconceptions about these necessities are prevalent among young athletes. This study investigated nutrition and hydration knowledge and practices among 28 male rugby union players aged 16 to 17, participating in Munster Rugby’s 2023 Summer Age-Grade Development Programme, specifically the U18′s Schools Squad. The Nutrition for Sport Knowledge Questionnaire assessed nutrition knowledge, while the Hydration Assessment Questionnaire evaluated hydration knowledge. Urinalysis for hydration status utilised urine specific gravity measurements pre-exercise on five separate days (1.018 ± 0.008 U_sg_). Dietary intake was recorded using a 3-day estimated food intake record on the Libro App, analysed with Nutritics software(Version 9.50). Suboptimal nutrition knowledge (49.6 ± 8.2%) and dietary practices were observed, with incongruent nutrient intakes compared to recommendations for adolescent athletes. While superior hydration knowledge (79.0 (77.3, 83.6) %) was evident, pre-exercise urine specific gravity readings indicated significant variation (*p* < 0.001) and signs of dehydration (USG > 1.020 U_sg_). No statistically significant correlations were found between knowledge and practices. The study highlights suboptimal nutrition and hydration knowledge and practices in youth athletes, suggesting the need for tailored support and educational interventions to enhance their overall health and performance. Further investigation into barriers and facilitators to dietary adherence is recommended for more effective interventions.

## 1. Introduction

Ensuring adequate energy, macronutrient and fluid intake is crucial to meet the various demands of sports [1]. The application of sports nutrition and hydration principles, grounded in scientific evidence, is instrumental in enhancing sports performance, supporting growth and maturation, facilitating recovery, and promoting the development of lean tissue mass, while minimising the risk of injury in adolescent athletes [2,3]. It is also important to recognise that nutritional choices have a significant impact on the physiological abilities and skillsets required in every sport, and specific to this current work, this includes rugby union (RU) [2].

Adolescence acts as a critical phase bridging the gap between childhood and adulthood, laying the foundations for long-term health [4,5]. It is concerning that many young individuals, particularly males engaged in rigorous training, commonly experience low energy availability and potential symptoms of relative energy deficiency in sport [1,6]. These issues can be further influenced by the differences in energy expenditure and requirements resulting from hormonal and biological variability in adolescents [6]. In particular, differences in the degree of maturation, such as peak height velocity, can result in significantly varied stature and mass in adolescent males of the same chronological age [6]. However, youth athletes often lack an awareness and understanding of the critical role that optimal nutrition and hydration plays in achieving overall health, well-being, and peak performance [2].

Previous studies have explored the nutritional knowledge of various groups of young athletes, including American high school soccer players [7], Division III Collegiate American athletes [8], and athletes from a wide range of school and collegiate sports (e.g., wrestling, soccer, volleyball, basketball, track and field, football, and tennis) [9]. Collectively, these studies have highlighted the need for improved sports nutrition and hydration knowledge among young athletes. This knowledge gap is also reported in adult athletes [10]. Furthermore, prior research has consistently demonstrated that young athletes often fail to adhere to the recommended dietary guidelines for their specific sport and activity level [1,6]. Correlations between dietary intake and nutritional knowledge have been explored in adult Australian soccer players [11], Australian rules football players [12], and Gaelic football and hurling players in Ireland [13,14]. The correlation identified in these studies indicate that athletes with stronger nutritional knowledge may be more inclined to apply this knowledge to their diets in a beneficial manner [10]. To the authors’ knowledge, no studies have been conducted to specifically investigate nutrition and hydration knowledge and practices concurrently in adolescent rugby players.

RU stands as one of the world’s most popular team sports, played across age groups and genders, with a considerable global following [15]. The professionalization of RU, beginning in 1995, has transformed the sport into a faster and more physically demanding game, largely due to rule changes and increased training demands [16]. The nature of RU, characterised by dynamic field-based play and intense physical confrontations, requires high levels of anaerobic and aerobic fitness, power, and strength in athletes to execute a range of actions, from sprinting to tackling [17,18]. RU positions are categorised into forwards and backs, with specialised front-row positions necessitating greater body and lean tissue mass to fulfil specific game-based tasks [19].

Physical attributes tend to vary significantly between elite, sub-elite, and youth RU players [17,18]. Numerous studies have examined the anthropometric and physical characteristics of adolescent RU players [18,19,20,21,22]. It has been suggested that young RU players could derive significant benefits from enhancing their absolute strength and power by increasing their lean tissue mass and maximising their force production capacity, as this is crucial for becoming a senior elite-level RU player [23]. Consequently, it is recommended that age-grade rugby players adopt a more individualised approach to their dietary intake due to RU’s unique demands and the greater emphasis on size demanded in professional rugby [16].

While there is a recognised need for increased lean tissue and body mass in the developmental pathways for rugby professionalism, there is a notable gap in the literature concerning the nutrition and hydration knowledge and practices of age-grade rugby players. Existing research has highlighted a lack of understanding regarding the foods and fluids necessary for effective refuelling, optimising sports performance, and the significance of protein in muscle development within an Irish cohort [24]. Walsh et al. [24] also highlighted a high level of disagreement among youth RU players regarding whether heavier players with greater lean tissue mass are more successful than lighter players in RU. Drawing definitive conclusions from this study is not possible due to the use of an unvalidated questionnaire [10]. Therefore, the findings from Walsh and colleagues’ [24] study merit a reassessment for adolescent Irish RU players, also due to evolving nutritional knowledge over the past 12 years [1].

Previous studies have assessed dietary intakes and diet quality in youth RU players, in Australia [25], England [26], and New Zealand [27,28]. Using validated methods and tools, these studies found that athletes generally complied with recommended energy and macronutrient intake percentages and achieved ‘good’ diet quality scores compared to national recommendations [25,26,27]. However, participants’ carbohydrate intake fell below recommended levels in comparison to specific youth athlete dietary guidelines (5–7 g·kg^−1^·day^−1^) [3]. This is particularly significant as insufficient carbohydrate intake can lead to fatigue, suboptimal decision-making, poor skill execution, and reduced concentration in athletes [2]. Roberts et al. [28] conducted a team-based case study with provincial academy RU athletes, where the research team attempted to facilitate improved nutrient intake by implementing a behavioural change protocol through education. The researchers observed increases in energy and macronutrient intakes following the provision of nutritional education. Notably, the nutritional knowledge of participants was not thoroughly assessed in any of these studies. Despite the importance of fluid intake and fluid replacement for daily bodily functions and pre-, during, and post exercise [29,30], hydration knowledge and practices were not investigated in any of these studies. There is a dearth of hydration-specific research in youth RU.

International rugby unions have devised strategic plans to foster the sport’s growth, with a central focus on Long-Term Athletic Development (LTAD) pathways [21,22,28]. These pathways aim to systematically nurture athleticism in young athletes, facilitating their safe participation in sports [31]. LTAD pathways expose young athletes to professional coaching, facilities, strength and conditioning programs, and the skills and knowledge needed for future opportunities within RU or elsewhere [28]. These pathways also strive to enhance player retention and bridge the transition from junior to elite senior-level sports, exemplified by summer talent development programs within provincial organisations in the Irish Rugby Football Union (IRFU) [32].

Building athleticism during LTAD is multifaceted due to individualised growth influenced by biological maturation [6,33]. Practitioners should prioritise both physical and cognitive development and advocate for an integrated learning approach to LTAD [31]. However, challenges such as congested schedules, a lack of awareness, and the neglect of cognitive aspects, including nutrition and hydration education, can occur due to an overemphasis on sport-specific training [10,31,34]. Providing suitable nutrition and hydration support during the LTAD stage is a collective responsibility among sports organisations, coaches, parents, teachers, and athletes [6].

Eat2Compete is the IRFU’s nutritional programme aimed at age-grade players to promote healthy eating for rugby performance [35]. This initiative offers general nutrition and hydration guidance in the form of educational videos and factsheets, accessible to players through the IRFU’s website. Eat2Compete covers areas such as match day nutrition and nutritional strategies for recovery, provides healthy snack lists, and offers helpful suggestions for managing hydration. However, there are no published data on Irish RU age-grade players to inform specific macronutrient requirements or their corresponding dietary intakes. Considering the paucity of peer-reviewed research available on nutrition and hydration knowledge and practices among adolescent athletes, it is timely to investigate what this cohort knows and what they actually practice.

For young athletes to optimise their health, well-being, and sports performance, it is crucial that they understand the specifics of energy, macronutrient, and fluid requirements on a daily basis, and specific to a training stimulus. Furthermore, the extant literature cautions that there may not be a direct application of knowledge to reported habitual food intakes [10].

Therefore, the aim of this current work was to investigate nutrition and hydration knowledge among age-grade RU players within an Irish context. Additionally, we examined the associations between knowledge levels and habitual hydration status, as well as nutrition practices concerning energy, macronutrient, and fluid intakes.

## 2. Materials and Methods

### 2.1. Study Design

**Figure 1:**
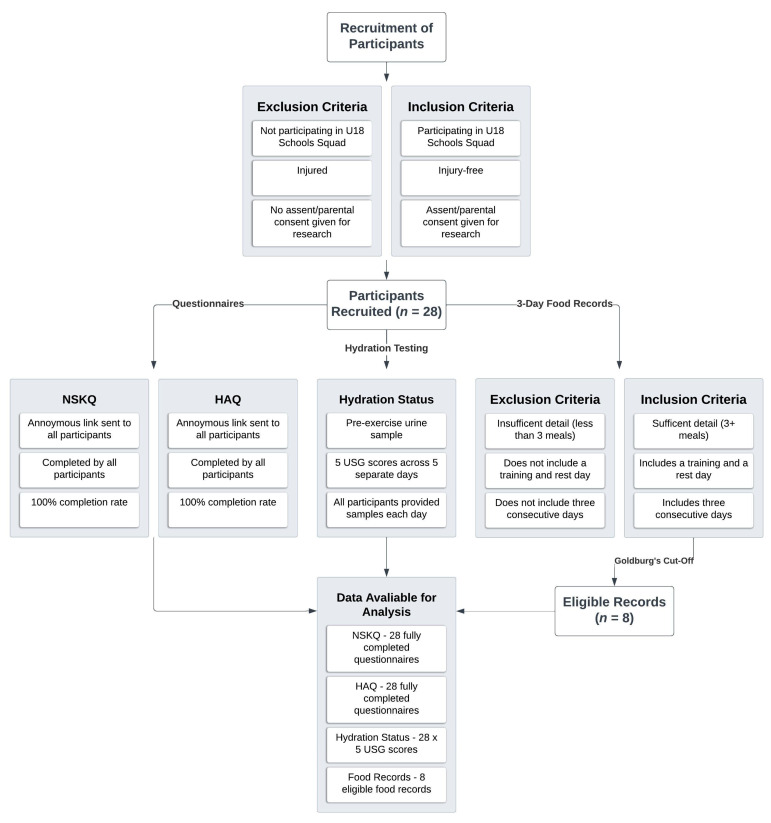
Selection and data collection process.

All of the male RU players (*n* = 28) participating in the under-18s Schools Squad during Munster Rugby’s Age-Grade Development Programme were invited to participate in this cross-sectional study. Data collection occurred over a two-week period in July 2023 at the development programme’s training base at the University of Limerick, Ireland. Figure 1 illustrates the selection process and the study design implemented.

### 2.2. Ethics

Ethical approval for this research was granted by the University of Limerick’s Education and Health Sciences Research Ethics Committee (2023_06_09_EHS). Comprehensive information regarding the study was provided to all participants and their parents/guardians through a participant information sheet. Subsequently, consent from parents/guardians and assent from all participating athletes were secured before the commencement of data collection.

### 2.3. Data Collection Protocol

Participant recruitment and consent procedures commenced at the onset of the summer program in June 2023. Details of the participants’ training schedule throughout the data collection period are provided in Table S1. Anthropometric measurements, the evaluation of participants’ hydration knowledge, and four pre-exercise urinalyses were conducted during the first week, coinciding with a residential training period where participants stayed overnight at the University of Limerick on Monday and Thursday. In the subsequent week, an additional pre-exercise urinalysis, an assessment of participants’ sports nutrition knowledge, and the collection of a 3-day estimated food intake record (eFIR) were performed. Week two was non-residential, with training sessions held at the University of Limerick and Musgrave Park, Cork. Continuous communication was maintained between the authors and participants during the data collection period.

### 2.4. Anthropometry

Anthropometric data were gathered from all participants at the beginning of the first morning during the initial week of data collection. Weight (kg) was recorded using electronic scales (Salter, Germany) to the nearest 0.1 kg, with participants wearing light clothing and ensuring empty bladders. Height (cm) was measured using a portable stadiometer (Seca, Leicester Height Measure) to the nearest 0.1 cm, while participants stood barefoot in the Frankfort plane position. All measurements were conducted by a trained investigator following the manufacturer’s instructions.

### 2.5. Knowledge Questionnaires

The extensively validated Nutrition for Sports Knowledge Questionnaire (NSKQ) [36] was employed to assess sports nutrition knowledge (File S1). The NSKQ comprises 89 questions separated into six subsections: weight management (12 questions), macronutrients (30 questions), micronutrients (13 questions), sports nutrition (13 questions), supplementation (13 questions), and alcohol (8 questions). A correct response was scored as +1 and an incorrect or ‘Not sure’ response was scored as 0. In line with previously published results, the interpretation of sports nutrition knowledge scores (%) was as follows: 0–49% (poor), 50–64% (average), 65–74% (good), and 75% or higher (excellent) [37]. The NSKQ was distributed to all participants following the completion of a full training day on day one of week two of data collection.

Hydration knowledge was assessed using the validated Hydration Assessment Questionnaire (HAQ) (File S2). This questionnaire was developed for a previous study investigating knowledge, attitudes, and behaviours regarding hydration and fluid replacement among collegiate athletes [38]. The HAQ is based on position statements released by the American College of Sports Medicine (ACSM) and the National Athletic Trainers Association (NATA). The questionnaire has demonstrated strong internal consistency, with Cronbach’s alpha levels for knowledge, attitude, and behaviour at 0.94, 0.92, and 0.96, respectively [38]. The HAQ consists of 51 questions, with 17 questions in each of the three subsections: knowledge, attitude, and behaviour. In the knowledge section, participants respond with ‘True’ or ‘False’. The attitude section requires participants to use a 5-point Likert scale, ranging from ‘Strongly Agree’ to ‘Strongly Disagree’, to answer the questions. In the behaviour section, participants respond with either ‘Yes’ or ‘No’. A correct response in the knowledge and behaviour sections was scored as + 1 and an incorrect response as 0. The questions in the attitude section were scored on the 5-point Likert scale, with a maximum score of 5 and a minimum score of 1. Results from the HAQ were compared to results from previous studies which also employed this questionnaire [38,39]. The lead researcher failed to receive a response from the HAQ author when attempting to obtain a scoring rubric for the questionnaire. Therefore, to maintain consistency between both questionnaires used in this study, the interpretation of results for the HAQ followed that of the NSKQ. The HAQ was distributed to all participants following the completion of a full training day on day two of the first week of data collection.

The questionnaires were hosted and distributed using Qualtrics survey application software (Version 07/2023, Qualtrics, Provo, UT, USA). Respondents were requested to complete both questionnaires independently. The anonymous responses were securely stored on the University of Limerick’s encrypted, password-protected data server. Demographic questions, such as age, playing position, school attended, completed school year in the previous academic year, and prior nutrition and hydration education, were adapted to suit Irish age-grade RU players.

### 2.6. Dietary Data Collection

Though lacking a universally accepted gold standard for quantifying energy intake, the predominant dietary assessment technique employed in sports nutrition research and practice is the food intake record [40,41]. This method requires participants to meticulously document all food and fluid consumption over a specified period, typically ranging from three to seven days. All participants were invited to complete a 3-day eFIR using the Libro mobile application (Version 9.50, Nutritics Ltd., Dublin, Ireland) to assess their habitual dietary intake, including energy, macronutrients, and fluids. Participants were instructed to maintain their eFIR over three consecutive days, including at least one training day and one rest day.

Instructions were provided to participants before commencing their eFIRs, guiding them on downloading, setting up, and utilising the Libro App (File S3). Invitations were sent via email, granting access for participants to download the app on their mobile devices. Once installed, users were automatically linked to a semi-structured 3-day eFIR within the app. To aid participants, the app offered online video tutorials, daily user instructions, and reminders for record maintenance.

Participants were required to log all food and fluid intake, specifying portion sizes, the time of consumption, and cooking methods (if applicable) for each meal or snack consumed during the 3-day recording period. Entries ranged from individual items (e.g., a banana, a bagel, or a cup of water) to composite items (e.g., a slice of pizza with toppings, a serving of spaghetti Bolognese, or yogurt with frozen berries and peanut butter). The app facilitated the accurate identification of food items either by manual input, where the app suggested options, or by barcode scanning, linked to a database aligned with European Union Reference Intake values [42].

Once an item was identified, users selected an estimated serving size, specified consumption times, and included any relevant cooking method notes. Upon finalisation, the item was saved and added to the participant’s record. Participants were instructed to maintain their usual dietary habits during the 3-day recording period, ensuring a reflection of their typical fuelling and energy consumption patterns.

### 2.7. Dietary Data Analysis

Following the completion of the 3-day eFIR, dietary intake data were exported from Nutritics (Version 9.50, Nutritics Ltd., Dublin, Ireland) for further analysis. A rigorous screening process ensured data completeness, requiring records spanning three consecutive days, encompassing both training and rest days while capturing comprehensive meal details. Accuracy checks were meticulously performed, addressing any discrepancies or seeking clarifications from participants as needed. The dietary analysis, encompassing energy, macronutrient, and fluid intake, was conducted using Nutritics. The resulting data were subsequently compared against sports nutrition recommendations (SNRs) for young athletes [3,43] and the Dietary Reference Values (DRVs) established by the European Food Safety Authority (EFSA) [42].

#### Misreported Dietary Records

Prior to conducting comparisons between eFIRs and SNRs and DVRs, adequately completed records underwent further screening to identify any misreporting of dietary intake. The revised Goldberg cut-off [44] was applied at both the group and individual levels to assess nutritional data within this subgroup of records. This process aimed to identify under-reporters (URs) and over-reporters (ORs), ultimately determining eligible reporters (ERs), i.e., records which were eligible for nutritional analysis.

The formulas used for assessing energy intake reporting were as follows:

For URs:

(1) $${EI}_{rep}:RMR>PAL\times exp\left[\;{SD}_{min}\times \frac{\left(S/100\right)}{\surd n}\;\right]$$

For ORs:

(2) $${EI}_{rep}:RMR<PAL\times exp\left[\;{SD}_{max}\times \frac{\left(S/100\right)}{\surd n}\;\right]$$

where EI_rep_ represents reported individual energy intake or the average of participants at the group level; physical activity level (PAL) was the mean PAL for participants. A PAL value of 1.9 was selected for the participants in this study. This value aligns with the mean PAL recommended for estimating energy requirements (EERs) in adolescent athletes [45]. *SD_min_* was set to −2 and *SD_max_* to +2 (95% confidence limits), and *n* was the number of participant records included (at the individual level, *n* = 1, at the group level, *n* = 10) [44]. S represents the factor that accounts for variation in intake, resting metabolic rate (RMR), and energy requirements, and was calculated as follows:

(3) $$S=\sqrt{\frac{{CV}_{wEI}^{2}}{d}+{CV}_{wB}^{2}+{CV}_{tP}^{2}}$$

where *CV_wEI_* represents intra-subject variation in energy intake (23%), *CV_wB_* was estimated to measured RMR precision (7.5%), *CV_tP_* was inter-subject PAL variation (15%), and d represents days of dietary assessment (3) [44]. The use of equations designed for adults to estimate RMR in adolescent athletes is discouraged. Research indicates that such equations tend to underestimate energy expenditure by as much as 300 kcal·day^−1^ when compared to measurements obtained through indirect calorimetry [6]. Therefore, RMR for all participants was calculated using the updated equation developed by Reale and colleagues [46]. This equation exhibits greater accuracy and reduces bias when compared to previous predictive models used to calculate RMR in adolescent athletes [6,46].

The formula for males is as follows:

RMR = 11.1 × Body Mass (kg) + 8.4 × Height (cm) − 340(4)

At a group level, cut-off was calculated at >1.65 for URs and <2.17 for ORs. At an individual level, cut-off was calculated at >1.23 for URs and <2.9 for ORs. Only ERs were included in the statistical analysis.

### 2.8. Hydration Status

Urine specific gravity (USG) served as the metric for evaluating participants’ hydration status. Pre-exercise urinalysis was assessed five times across the 2-week data collection period, utilising a calibrated digital refractometer (Atago 3741 (PEN-Urine S.G.), Cole Palmer, UK). The measurement accuracy of the PEN-Urine S.G. refractometer is ±0.001 U_sg_ [47], with demonstrated reliability in assessing hydration status [48]. Participants were instructed to maintain their habitual hydration strategies throughout the assessment period.

Morning mid-stream urine samples (approximately 10 mL) were collected upon arrival at the training base between 8:30 a.m. and 10:30 a.m., following the schedule outlined in Table S1. Samples were promptly analysed upon collection, with the refractometer calibrated using distilled water and cleaned between samples. Recalibration was undertaken after every three samples [13]. To obtain a reading, the refractometer’s tip was immersed approximately an inch into the urine sample, activating measurement by pressing the ‘start’ button until a value appeared. Subsequent to recording measurements, urine samples were disposed of appropriately. Dehydration was defined as a USG reading exceeding 1.020 U_sg_ [29]. Environmental conditions (temperature, humidity, and time) during sample collection were recorded using forecasts from the Irish Meteorological Service website [49].

### 2.9. Statistical Analysis

Statistical analyses were conducted using SPSS (Version 28, IBM Corp., Armonk, NY, USA), with a significance level set at *p* < 0.05, employing two-tailed tests. To assess the normality of the data, Shapiro–Wilk testing was applied. Parametric data are presented as means ± SD, while non-parametric data are presented as medians and interquartile ranges (IQR). Participants were categorised into knowledge groups (poor, average, good, or excellent) for both questionnaires, hydration status (hydrated (USG ≤ 1.020), significantly dehydrated (USG 1.021–1.030), or severely dehydrated (USG > 1.030)) [50], and energy intake (meeting or not meeting recommendations). This ordinal categorization facilitated the examination of associations between knowledge and practice in the analysis.

ER NSKQ scores and their reported energy, macronutrient, and fluid intake data were transformed to ordinal data to allow for correlations between these elements to be explored using Spearman’s rank-order correlation. Due to normally distributed scores, one-sample *t*-tests compared total and subsection NSKQ scores of the whole cohort against the scoring criteria. Additionally, one-sample *t*-tests were conducted to compare the normally distributed reported energy and macronutrient intakes of ERs to established SNRs for youth athletes [3,43] and DRVs for nutrients by the EFSA [42].

Somers’ *d* was utilised to identify associations between hydration knowledge and practice. Due to non-normally distributed scores, one-sample Wilcoxon signed-rank tests compared total and subsection HAQ scores of the whole cohort against the NSKQ scoring criteria. These tests were also used to compare USG scores from each testing day to the cut-off point to signify dehydration (USG > 1.020) as normality was not present in these daily scores. Additionally, Friedman’s two-way analysis of variance was employed to examine the distributions of USG scores across the five days, while the Wilcoxon signed-rank test was utilised to evaluate differences between the days with the highest and lowest instances of dehydration.

## 3. Results

### 3.1. Participant Demographics

Participant characteristics are summarised in Table 1. The study included twenty-eight participants aged between 16 and 17 years, who had completed either the fourth or fifth year of secondary school in the previous June prior to data collection. Ten different schools across the province of Munster were represented in the cohort. All playing positions in RU were represented in the sample. Additionally, all participants indicated having received or completed prior nutritional education.

### 3.2. Outcome Measures

Both knowledge questionnaires achieved a 100% completion rate in this study. Of the submitted eFIRs, ten were deemed adequately completed after thorough screening for completeness. The application of Goldberg’s cut-off [44] to these records led to the exclusion of two from the subsequent statistical analysis due to identified underreporting of dietary intake. All twenty-eight athletes provided a mid-stream urine sample each day during the urinalyses.

#### 3.2.1. Nutrition for Sports Knowledge Questionnaire Results

Twenty-eight athletes completed the NSKQ (Table 2). The mean total NSKQ score for the sample (49.6 ± 8.2%) fell within the “poor” nutrition knowledge category (0–49%), which was not significantly below the threshold for “average” nutrition knowledge (*p* = 0.818). The majority of participants (57.1%, *n* = 16) exhibited “poor” nutrition knowledge, with the remainder were categorised as having “average” (35.7%, *n* = 10) and “good” (7.1%, *n* = 2) nutrition knowledge (50–64% and 65–74%, respectively).

The Supplements subsection (36.5 ± 12.8%) scored significantly lower than the threshold for “average” nutrition knowledge (*p* < 0.001). Additionally, the Micronutrients (44.6 ± 18.8%) (*p* = 0.050) and Sports Nutrition (46.7 ± 12.1%) (*p* = 0.170) subsections were categorised as “poor” in terms of nutrition knowledge, with no significant difference noted. The Weight Management (53.8 ± 12.1%) (*p* = 0.130), Macronutrients (55.0 ± 12.4%) (*p* = 0.043), and Alcohol (59.4 ± 16.5) (*p* = 0.006) subsections were categorised as “average” at the group level. All subsection scores were below the criterion for “good” and “excellent” (>75%) nutrition knowledge.

#### 3.2.2. Hydration Assessment Questionnaire Results

Twenty-eight athletes completed the HAQ (Table 2). The median total HAQ score for the sample was 79.0 (77.3, 83.6) %, indicating “excellent” hydration knowledge at the group level (*p* = 0.007). A majority of participants (78.5%, *n* = 22) scored ≥ 75% in the HAQ, ranging from a high of 89.1%, achieved by two respondents, to a low of 57.1%, obtained by one responder. The median knowledge (88.2 (76.5, 92.6) %) (*p* < 0.001) and attitude (78.8 (75.3, 83.2) %) (*p* = 0.008) scores surpassed the threshold for “excellent” hydration knowledge, whereas the median behaviour score (76.5 (70.6, 82.4) %) did not significantly differ from the “excellent” level (*p* = 0.360). Attitude and behaviour scores were significantly lower than the knowledge score at the group level (*p* = 0.003, *p* ≤ 0.001). Three athletes (10.7%) achieved a perfect knowledge subsection score of 100% (17/17), whereas no participant attained a perfect score in the attitude and behaviour subsections.

#### 3.2.3. Energy, Macronutrient, and Fluid Intake

All participants were invited to complete a 3-day eFIR, resulting in the return of fifteen records, which underwent preliminary screening for inclusion (Figure 1). Ten eFIRs met the inclusion criteria for eligibility and were subjected to Goldberg’s cut-off [44] to identify potential misreporting and determine their eligibility for statistical analysis. At the group level, participants were classified as URs. No participant fell into the OR category at the individual level.

However, two participants were categorised as URs and eight as ERs. The average energy and macronutrient intake for participants defined as ERs (*n* = 8) was compared to both SNRs [3,43] formulated for adolescent athletes and European Union DRVs [42] (Table 3).

Reported energy intakes were significantly lower than estimated requirements, as evidenced by 87.5% (*n* = 7) of athletes experiencing an energy deficit of 693 ± 663 kcal·day^−1^ compared to their individual EER. No ER adhered to the 5–7 g·kg^−1^·day^−1^ carbohydrate recommendations suitable for 1 h of skills-based or moderate-intensity daily exercise, with one ER exceeding and the remaining ERs failing to meet this recommendation. Similarly, average carbohydrate intake failed to meet the recommended percentage of total energy intake, with 37.5% (*n* = 3) of athletes reaching this target. However, two of these ERs failed to meet their individual EER.

The ERs reported protein intake exceeded the recommended guidelines, with 75% (*n* = 6) of athletes consuming ≥2 g·kg^−1^·day^−1^. However, its distribution across meals was uneven, notably with breakfasts displaying the lowest and dinners the highest protein content among ERs. Additionally, the use of a protein supplement was reported in a single food record. Average fat intake was within the recommended range for adolescent athletes, although 37.5% (*n* = 3) consumed ≥35% of their total energy intake from fats. Inadequate fluid consumption was noted in 25% (*n* = 2) of athletes, while the remaining athletes exceeded the recommended fluid intake, averaging ≥3375 mL·day^−1^.

#### 3.2.4. Urine Specific Gravity Scores

**Figure 2:**
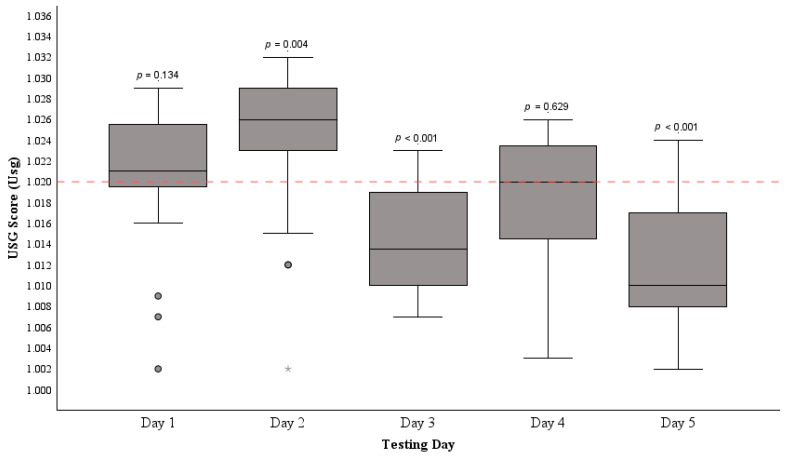
The distribution of median USG scores among age-grade RU players (*n* = 28) before exercise across five separate testing days. The horizontal dashed line at 1.020 indicates the dehydration threshold. The mean USG over the five days significantly differed from the dehydration cut-off, as confirmed by a one-sample *t*-test (*p* = 0.011). Median USG was compared to the dehydration threshold each day with significance indicated by *p*-values. • represent IQR outliers and * represents an extreme IQR outlier.

The pre-exercise hydration status of participants (*n* = 28) is displayed in Figure 2. The mean USG (1.018 ± 0.008 U_sg_) recorded across the five testing days was below the cut-off point to signify dehydration (USG > 1.020) (*p* = 0.011). The highest instances of dehydration occurred on day one (1.021 (1.019, 1.026) U_sg_) (*p* = 0.134), with 64.2% (*n* = 18) of participants, and day two (1.026 (1.023, 1.029) U_sg_) (*p* = 0.004) with 82.1% (*n* = 23) of participants returning urine samples indicating dehydration. On day four, 46.4% of athletes (*n* = 13) returned samples signifying dehydration (1.020 (1.014, 1.024) U_sg_) (*p* = 0.629). Days three (1.014 (1.010, 1.019) U_sg_) (*p* < 0.001) and five (1.010 (1.008, 1.017) U_sg_) (*p* < 0.001) saw a minimum of 14.2% (*n* = 4) of athletes exhibiting dehydration prior to exercise. Throughout the testing period, temperatures ranged from 12 to 17 °C, with humidity levels between 77 and 94%.

Friedman’s two-way analysis of variance by ranks was utilised to assess the similarity of USG score distributions across the five testing days, revealing a notable discrepancy among the distributions of USG scores across different days (*p* < 0.001). Subsequent analysis through the Wilcoxon signed-rank test compared the average USG scores between the days with the highest prevalence of dehydration (Day 1 and 2) against those with lower prevalence (Day 3, 4, and 5). This comparison suggests a significant difference between these pooled score groups (*z* = −4.184, *p* < 0.001).

#### 3.2.5. Associations between Knowledge and Practice

A Spearman’s rank-order correlation was conducted to assess the relationship between ER NSKQ scores and their energy, macronutrient, and fluid intakes. The correlations ranged from −0.455 to 0.333, signifying moderate negative to weak positive associations, none of which were statistically significant (*p* > 0.05). The absence of statistical significance indicates no clear linear relationship between overall nutrition knowledge and dietary intake elements within this study’s ERs. This suggests that the measured nutrition knowledge via the NSKQ might not directly influence or strongly correlate with daily dietary intake elements in this dataset.

Employing Somers’ *d*, the association between HAQ and USG 5-day average scores among the twenty-eight participants was examined, uncovering a very weak, non-significant positive association (*d* = 0.67, *p* = 0.694). This implies a limited connection between hydration knowledge assessed in the HAQ and the actual hydration practices of the whole cohort in this study.

## 4. Discussion

To the authors’ knowledge, this study represents the first investigation of nutrition and hydration knowledge of age-grade RU players while concurrently examining their practices, reported energy, macronutrient, and fluid intake, as well as measured hydration status. The key finding is an inadequate sports nutrition knowledge among this specific cohort, relative to that necessary to support optimal health, wellbeing, training, and performance. While the dietary assessment was limited to a subgroup who provided eligible food records, it is evident that their habitual nutrition practices were also suboptimal. We report an inadequate intake of dietary energy, particularly from carbohydrates, coupled with an excessive intake of protein and, in some cases, fat. Despite participants displaying a higher level of hydration knowledge, in practice, their actions did not align until after the completion of the HAQ. This discrepancy suggests that a potential learning effect possibly occurred over the urinalysis period in this study.

The observed lack of nutrition knowledge among the age-grade RU players in this study may have influenced their dietary intake, as suggested by weak-to-moderate correlations between NSKQ scores and players’ intake of energy, macronutrients, and fluids. Notably, none of these correlations were statistically significant (*p* > 0.05). Comparing the nutrition knowledge score (49.6 ± 8.2%) in this study to previous research conducted in Ireland, it is lower than that found in 15 to 18-year-old adolescent RU players competing at Leinster Schools Senior Cup level (59.6 ± 12.8%) [24] and adult club and university athletes, including RU participants (52.9 (46.0, 59.8) %) [51]. These comparisons are constrained by the diversity of sampling methods used. When compared to other athletic groups using the same tool, this cohort demonstrated slightly better performance than elite adult male Australian rules footballers (45.5 ± 14.7%) [37] adult male Gaelic footballers (40.2 ± 12.4%) [13], and male and female American collegiate athletes (47.9 ± 11.3%) [8]. All cohorts assessed through the NSKQ for their nutrition knowledge were categorised as having “poor” nutrition knowledge, indicating a widespread issue, ubiquitous across countries, sports, athletes, ages, and genders, that necessitates corrective action.

In athletic populations, discrepancies between reported and actual food and fluid intake are common [41]. Several factors, such as participant burden, motivation, willingness to accurately report diet, and data entry errors, can introduce systematic errors in assessing dietary habits [40,52]. The use of dietary assessment applications, including mobile phone apps, has shown promise in increasing participant engagement and satisfaction compared to traditional methods [52,53]. To enhance engagement, the present study employed the Libro App. Despite this approach, only eight eFIRs were eligible for statistical analysis. The energy intakes observed in the present study (3456 ± 740 kcal·day^−1^) were marginally higher than values reported in RU academy players in England (3412 ± 670 kcal·day^−1^) [26] and New Zealand (2614 ± 625 kcal·day^−1^) [28]. However, even with the exclusion of URs, the energy intake of ERs fell short of meeting their EER, resulting in a substantial average daily energy deficit of 693 ± 663 kcal·day^−1^. When aiming to optimise the performance of adolescent athletes through nutrition, ensuring adequate calorie consumption is of the greatest concern [43]. Therefore, acknowledging the potential for under-reporting, sporting organisations should intensify efforts to enhance the energy intake of future participants in talent development programs. It is crucial for practitioners to highlight the significance of providing players with consistent access to regular meals and snacks, enabling adequate energy supply for growth, development, and adaptations during such talent development programs.

Insufficient energy intake in this cohort appears to be predominantly linked to inadequate carbohydrate consumption (Table 3), consistent with findings from previous studies on adolescent RU players [26,27]. The low intake of carbohydrate among ERs might stem from poor knowledge or misguided beliefs, as indicated in responses to carbohydrate-specific questions in the NSKQ. Notably, 62.5% (*n* = 5) of ERs incorrectly identified carbohydrates as having the highest energy content (kilojoules/calories) per 100 g among the three macronutrients, in contrast to 82.1% (*n* = 23) in the overall group. One potential explanation for this belief is the prevailing public perception, often amplified by popular media, that carbohydrate-rich foods are unhealthy [25]. Confusion regarding the utilisation of lower glycaemic index carbohydrates for appetite control was evident, with 75% (*n* = 6) of ERs responding as “Not sure” about this carbohydrate group’s efficacy for weight loss, compared to 46.4% (*n* = 13) who correctly answered this question within the entire cohort. Consequently, participants may avoid this macronutrient, fearing weight gain and performance decline [54].

Differences in comprehension regarding carbohydrate utilisation for recovery after high-intensity aerobic exercise were also evident among ERs. For instance, while the majority deemed consuming one medium-sized banana (87.5%, *n* = 7) or one cup of plain yogurt (75%, *n* = 6) inadequate, there was a consensus (87.5%, *n* = 7) that consuming one cup of baked beans on two slices of bread was sufficient. However, uncertainty surrounded the adequacy of a recovery meal comprising one cup of cooked quinoa and one tin of tuna, with 50% (*n* = 4) of ERs considering it “Enough”, 37.5% (*n* = 3) responding as “Not sure”, and one ER correctly indicating “Not enough”. Additionally, a single ER correctly answered the question regarding the ideal carbohydrate intake per kilogram for an athlete engaging in moderate- to high-intensity endurance training for two hours, a question answered accurately by only 35.7% (*n* = 10) of the entire participant group. The recent surge in popularity of ‘low-carb’ diets and an excessive emphasis on leanness in sports resulting in body image issues might contribute to this confusion in young athletes [6]. This approach of low carbohydrate consumption is counterintuitive for team sport athletes, particularly RU players. These athletes require high levels of physicality and cognitive function for optimal performance, both of which can be impaired by inadequate carbohydrate consumption [2].

Research has consistently shown that youth athletes tend to consume protein in quantities that surpass SNRs [3,43] and DRVs [42], a trend also observed in this study. Protein intake within ERs was notably high but not evenly distributed across meals. Reported breakfasts exhibited the lowest mean protein content (36.4 ± 12.5 g), followed by lunches (43.4 ± 19.4 g), while dinners showed the highest protein content (60.6 ± 20.5 g) among the ERs investigated. The NSKQ results reveal differing responses concerning the body’s limited ability in the utilisation of protein for muscle protein synthesis, with 50% of ERs either disagreeing (25%, *n* = 2) or being “Not sure” (25%, *n* = 2) about the accuracy of this statement. At the group level, 53.5% (*n* = 15) answered this question correctly, while 14.2% (*n* = 4) disagreed, and 32.1% (*n* = 9) were “Not sure”. Although 50% (*n* = 4) of ERs correctly answered the question related to this concept, their dietary practices did not align with this knowledge, as their protein intakes varied widely within daily meals. This is a common phenomenon across athletic and general populations [55]. Athletes are advised to aim for a 30 g bolus of high-biological-value protein per meal, representing the optimal threshold to initiate muscle protein synthesis. However, on average, the 30 g threshold is exceeded at each main meal within this cohort, suggesting a potential bias toward these meals. Furthermore, this practice has been linked to a reduction in unhealthy snacking habits among younger individuals [55], behaviours previously reported in youth RU athletes [26,27] and also evident in the present study.

The lack of clarity regarding appropriate protein intake is further emphasised by all ERs providing incorrect responses in relation to the recommended amount of protein an athlete should consume after resistance exercise, with 10.7% (*n* = 3) of the entire cohort answering this question accurately. The human body is incapable of storing surplus protein and instead oxidises it [54]. Consequently, consuming large amounts of protein per meal is considered suboptimal practice which offers no adaptive advantage. Moreover, excessive protein intake may lead to a displacement of carbohydrate intake due to suppressed appetite and increased satiety [55]. Among the studied ERs, a single individual who reported consuming ≥2 g·kg^−1^·day^−1^ of protein slightly exceeded the recommended carbohydrate intake for adolescent athletes (5–7 g·kg^−1^·day^−1^), consuming 7.2 g·kg^−1^·day^−1^ of carbohydrate. Conversely, the remaining ERs who consumed excessive amounts of protein (62.5%, *n* = 5) reported a consumption of 4.2 ± 0.4 g·kg^−1^·day^−1^ of carbohydrate, failing to meet SNRs and their individual EERs.

Parallel misconceptions were evident regarding fat intake and, specifically, fat oxidation within energy pathways. Among ERs, 75% (*n* = 6) believed that as exercise intensity increases, the percentage of fat used for fuel also increases. Three of these athletes had a fat intake exceeding recommendations for youth athletes (>35% EI) [3]. This belief might explain why these athletes had a higher than recommended fat intake, with an additional two nearing the upper limit of the recommended intake (>30% EI). Similarly, all ERs either disagreed (62.5%, *n* = 5) or were “Not sure” (37.5%, *n* = 3) in regard to fat being the primary fuel source during low-intensity exercise. Once again, 10.7% (*n* = 3) of the entire cohort provided accurate responses to this question. This is concerning as human metabolism relies primarily on the oxidation of carbohydrates as its fuel source during high-intensity exercise [43]. It is probable that the participants in this study are unaware of the specific SNRs for energy and macronutrient intake set forth by international sporting committees [3,54]. Limited knowledge is leading to a restricted ability to apply SNRs to their dietary habits [34]. As a result, age-grade RU players in this study report an inadequate diet for demands related to training and performance.

The overall HAQ scores (79.0 (77.3, 83.6) %) of this cohort surpassed those of American collegiate athletes (69.6 ± 12.7%) [38], with each subsection score also exhibiting higher results in the present study. The hydration knowledge (88.2 (76.5, 92.6) %) of this cohort was greater than that of Irish adolescent RU players previously studied, albeit using a different tool but with similar questions (76.4 ± 20.7%) [24]. The average score in the knowledge subsection similarly outperformed a more recent study using the HAQ on American collegiate football players (69.4 ± 11.2%) [39]. A comparison between the attitude and behaviour subsections within this study cannot be made as these results were not explicitly reported.

Participants exhibited a high level of general hydration knowledge practices, acknowledging the necessity of fluid intake during training, the significance of easily accessible fluids during exercise, and the impact of alcohol consumption on dehydration before training or competition. However, knowledge statements concerning ACSM and NATA position stands, particularly regarding the correct use of sports drinks, were frequently answered incorrectly. Fewer than half of the participants (46.4%, *n* = 13) recognised that athletes should consume sports drinks during exercises lasting over an hour, while 35.7% (*n* = 10) disagreed on the necessity of drinking 500–600 mL of water/sports drink in the hours preceding competition. Furthermore, 17.9% (*n* = 5) expressed disbelief in the consumption of 200–300 mL of water/sports drink 10–20 min before competition. Additionally, 46.4% (*n* = 13) did not endorse the superiority of sports drinks over water for replenishing glycogen in muscles. Only two ERs reported consuming a sports drink on a training day, and both ERs answered all questions related to sports drinks correctly. This once again highlights a lack of knowledge and application of sports specific guidelines in this study’s participants. Similar response trends in relation to ACSM and NATA position stands have been reported previously [38,39,56]. These knowledge gaps regarding hydration strategies pre-, during, and post exercise are concerning as they could potentially impact performance and lead to health issues during intense physical activity [29].

The attitude and behaviour subsection scores of the HAQ were notably lower than the knowledge subsection. This discrepancy implies that while there is adequate hydration knowledge, its translation into practical hydration habits is lacking, not only in this cohort but also in previous studies [39,56]. This is reflected in the initial two days of urinalysis. Despite the majority of athletes (96.4%, *n* = 27) indicating that they rely on urine colour to assess hydration status, most of these individuals exhibited dehydration in urine samples on day one (60.7%, *n* = 17) and day two (71.4%, *n* = 20) of urinalysis, with an additional two showing severe dehydration (USG > 1.030) on day two. Likewise, 75% (*n* = 21) of participants claimed to consume 500–600 mL of fluids before exercise, yet a majority were dehydrated prior to exercise on day one (46.4%, *n* = 13) and day two (64.2%, *n* = 18). Only one athlete claiming adequate pre-exercise fluid intake returned urine samples indicating appropriate hydration on both days. For most participants, this disparity indicates a gap between their knowledge and practiced behaviour. Notably, following the distribution of the HAQ on the evening of day two of urinalysis, hydration practices improved. Only 14.2% (*n* = 4) of athletes produced samples indicating dehydration on days three and five. However, on day four, 46.4% (*n* = 13) exhibited pre-exercise dehydration. Yet, the high *p*-value (*p* = 0.629) suggests that these samples were not significantly different from the dehydration threshold. Hence, it is likely that the enhancement in USG scores after completing the HAQ was influenced by a learning effect among participants.

The 5-day average USG score in this cohort (1.018 ± 0.008 U_sg_) was higher than in previous Irish studies that investigated pre-exercise hydration status: (1.015 (1.007, 1.022) U_sg_) [51]; (1.010 (1.005, 1.017) U_sg_) [13]. Comparing these studies presents challenges as they conducted pre-exercise urinalysis only once with their respective participants. Additionally, the potential learning effect observed in the present study further complicates direct comparisons. However, additional research on age-grade RU players’ hydration status and practices, employing validated measures both before and after exercise, is necessary. It is crucial to educate young RU players about commencing exercise adequately hydrated, strategies for maintaining hydration during exercise, and appropriate strategies for rehydration post exercise. Implementing individualised hydration plans for young RU players is advisable to mitigate the risk of dehydration and its potential health and performance consequences [29]. Incorporating water breaks during training and utilising halftime and breaks in play during competition could help ensure adherence to these personalised hydration plans.

This study is not without its limitations. The data collection occurred within a constrained timeframe, integrated into the summer development program with minimal disruption to the players as per their coaches’ request. Despite achieving a 100% completion rate for both questionnaires, independent completion by participants cannot be guaranteed due to their digital dissemination and completion.

A lack of engagement with dietary recording and under-reporting are common in dietary assessments [41] and were evident in this study. To address and mitigate these issues, specific efforts were undertaken, including providing clear instructions to ensure records included sufficient detail, the utilisation of the Libro App, maintaining constant communication with participants, and the application of Goldberg cut-offs [44] on returned food records. It should be noted that food records were the final element of the data collection process in this study. Consequently, the observed lack of engagement with this process may be attributed to research fatigue, leading participants to perceive dietary data collection as time-consuming and potentially detracting from their training or competition commitments [41].

Energy, macronutrient, and fluid values reported from food records should be interpreted with caution, as these values are estimates due to records not being weighted. EER, RMR, and PAL values were also estimates for this specific cohort and may not be a true representation to these values, as direct measurement was outside the scope of this study.

The urinalysis revealed a potential learning effect post HAQ completion; therefore, the hydration practices indicated afterward may not entirely mirror participants’ typical habits and should be cautiously interpreted. Using an updated questionnaire to assess hydration knowledge has been recommended [10]. Given the small sample size, any extrapolation of results should be approached with caution.

Future studies should structure data collection to avoid overburdening participants and introducing potential biases. Timing for questionnaire dissemination should be appropriate to prevent interference with other aspects of data collection, as well as training and performance commitments. Strong considerations should be made when undertaking dietary assessments with future participants. Suitable timing should be discussed with coaches and should also be fully supported by all stakeholders in order to improve participant engagement in this element of data collection.

## 5. Conclusions

In conclusion, our findings corroborate previously reported inadequate nutrition knowledge and practices among age-grade RU players. Suboptimal energy and carbohydrate intakes, falling short of recommendations for adolescent athletes, were evident. Despite some improvements in hydration knowledge, relative to nutrition, there is a notable lack of alignment with attitudes and claimed behaviour when compared to actual practices. Ensuring sufficient nutritional intake is crucial for enhancing peak performance, well-being, and development, while also protecting adolescent athletes from potential health issues related to dietary deficiencies. Recognising that nutrition and hydration knowledge are malleable aspects of dietary behaviour, it is essential to pursue enhancements. Future research should explore all modifiable factors influencing male and female adolescent athletes’ dietary intake. Sporting organizations should develop early screening strategies and provide continuous nutritional education interventions to youth athletes to ensure they possess the knowledge required for optimal practices for both their sport and for maintaining overall health. Additionally, allowing for individualised player support from qualified professionals, whenever feasible, should be a key consideration.

## Figures and Tables

**Table 1:** Demographic characteristics of age-grade RU players (*n* = 28).

Characteristic	Total Cohort (*n* = 28)	Under-Reporters (*n* = 2)	Eligible Reporters (*n* = 8)
Age (years)	17 ^$^ (16, 17)	17	17 (16, 17)
Weight (kg)	89.8 ± 11.5	97.0 ± 0.5	89.6 ± 12.5
Height (cm)	182.1 ± 7.0	188.5 ± 4.5	182.0 ± 9.4
RMR (kcal·day^−1^)	2187 ± 175 ^∞^	2320 ± 43	2183 ± 210
EER (kcal·day^−1^)	4155 ± 332 *	4408 ± 82	4148 ± 399
EI_rep_ (kcal·day^−1^)	3181 ± 880 ^£^	2080 ± 405	3456 ± 740
EI_rep_ to RMR ratio	1.45 ± 0.4 ^€^	0.89 ± 0.16	1.59 ± 0.32

kg kilograms, cm centimetres, RMR resting metabolic rate, EER estimated energy requirements, EI_rep_ reported energy intake, EI_rep_: RMR energy intake to RMR ratio, kcal day^−1^ energy per day. Ten eFIRs passed initial screening. Two eFIRs were identified as under-reporters and eight eFIRs were identified as eligible reporters upon the application of Goldberg’s cut-off. ^∞^ RMR = 11.1 × Body Mass (kg) + 8.4 × Height (cm)—340 [46]. ^$^ Values presented as median (interquartile range). * EER = RMR × PAL (1.9) [45]. ^£^ Average energy intake from food records, *n* = 10. ^€^ Average EI_rep_ to RMR from food records, *n* = 10.

**Table 2:** NSKQ and HAQ scores for age-grade RU players (*n* = 28).

NSKQ Scores (%)		*p*-Value *	HAQ Scores (%)		*p*-Value *
Overall	49.6 ± 8.2	0.818 ^a^	Overall	79.0 (77.3, 83.6)	0.007 ^b^
By subsection			By subsection		
Weight Management	53.6 ± 11.9	0.130 ^a^	Knowledge	88.2 (76.5, 92.6)	<0.001 ^b^
Macronutrients	55.0 ± 12.2	0.043 ^a^	Attitude	78.8 (75.3, 83.2)	0.008 ^b^
Micronutrients	44.6 ± 18.8	0.050 ^a^	Behaviour	76.5 (70.6, 82.4)	0.360 ^b^
Sports Nutrition	46.7 ± 12.1	0.170 ^a^			
Supplementation	36.3 ± 12.8	<0.001 ^a^			
Alcohol	59.4 ± 16.2	0.006 ^a^			

Criteria for performance in the questionnaires: 0–49% (poor), 50–64% (average), 65–74% (good), ≥75% (excellent). NSKQ scores are presented as mean ± SD. HAQ scores are presented as median (IQR) * *p*-values were determined using a one-sample *t*-test for NSKQ results and one-sample Wilcoxon signed-rank tests for HAQ results. ^a^ Score compared to an “average” threshold of 50% ^b^ Score compared to an “excellent” threshold of 75%.

**Table 3:** Average energy and macronutrient intake of age-grade RU players (*n* = 8).

Nutrient	Reported Intake (Mean ± SD)	EFSA DRV/SNR Recommended Target/Range	Athletes Meeting DRV or SNR Target/Range *n* (%) ^¥^	*p*-Value *
Energy				
kcal·day^−1^	3456 ± 740	4148 ± 399 ^d^	1 (12.5)	0.043
Carbohydrate				
g·day^−1^	384.6 ± 78.6	N/A	N/A	
g·kg^−1^·day^−1^	4.4 ± 1.2	5–7 ^a^	0 (0)	0.218
% EI	45.0 ± 7.2	> 50 ^b^	3 (37.5)	0.107
Protein				
g·day^−1^	194.8 ± 46.2	N/A	N/A	
g·kg^−1^·day^−1^	2.2 ± 0.5	1.3–1.8 ^a^	2 (25)	0.064
% EI	23.1 ± 4.2	15–20 ^b^	1 (12.5)	0.093
Fat				
g·day^−1^	125.5 ± 50.9	N/A	N/A	
g·kg^−1^·day^−1^	1.4 ± 0.5	N/A	N/A	
% EI	32.0 ± 7.9	20–35 ^ac^	5 (62.5)	0.345
Fluid				
ml·day^−1^	3360 ± 1142	2500 ^c^	6 (75)	0.087
ml·kg^−1^·day^−1^	39 ± 17	N/A	N/A	

DRV dietary reference value, SNR sports nutrition recommendation, kcal day^−1^ energy per day, g·day^−1^ g per day, g kg^−1^ day^−1^ g per kilogram per day, % EI percentage of energy intake, mL·day^−1^ millilitres per day, mL·kg^−1^·day^−1^ millilitres per kilogram per day, N/A not applicable, n participants. * *p*-value determined by one-sample *t*-test. Intake compared to DRV/SNR. Data compared to lower (carbohydrate g kg^−1^ day^−1^) or upper range (protein g kg^−1^ day^−1^ and % EI, fat % EI), where appropriate. ^¥^ Protein g kg^−1^ day^−1^ and % EI, carbohydrate g kg^−1^ day^−1^ and % EI, fat % EI (between ranges used) and energy intake compared to individual requirements. ^a^ Sports Dietitians Australia Position Statement [3]. ^b^ Nutritional considerations for performance in young athletes [43]. ^c^ European Food Safety Authority DVR [42]. ^d^ EER = RMR × PAL (1.9) [45].

## Data Availability

Data are contained within the article and Supplementary Materials.

## Supplementary Materials

The following supporting information can be downloaded at: https://www.mdpi.com/article/10.3390/nu16040533/s1, Table S1: Participants’ training schedule and data collection process; File S1: Copy of the Nutrition for Sport Knowledge Questionnaire; File S2: Copy of the Hydration Assessment Questionnaire; File S3: Copy of study background and Libro App information presentation.
