# Supplementary material for: Investigating Nutrition and Hydration Knowledge and Practice among a Cohort of Age-Grade Rugby Union Players
Source: Nutrients. 2024 Feb 14;16(4):533. doi: 10.3390/nu16040533 (PMC10891571; doi:10.3390/nu16040533)
Supplement: Supplementary file 1 [file nutrients-16-00533-s001.zip › File S1. Copy of The Nutrition for Sport Knowledge Questionnaire.pdf]

File S1. Copy of The Nutrition for Sport Knowledge Questionnaire

---

Start of Block: Introduction

Thank you for participating in this study entitled: Investigating Nutrition and Hydration Knowledge and Practice Among Age Grade Rugby Union Players. This questionnaire examines your nutrition knowledge and practices. It should take 15 to 20 minutes to complete.

Our aim for this research is to investigate nutrition and hydration knowledge and practice among age grade rugby union players participating in the Munster Rugby Age-Grade Player Development Programme. Associations with habitual hydration status and / or dietary practices (energy and macronutrient intakes) and sports performance (speed, strength, endurance) will be investigated.

Please read each question carefully and answer each question as truthfully as possible. If you do not know the answer to a particular question do not guess. Use the 'Not Sure' option for questions you do not know the answer to.

If you wish to ask any more questions about the research process you can contact the researchers at the following: Catherine Norton - catherine.norton@ul.ie or Shane Scanlon - 13158996@studentmail.ul.ie.

This research study has received Ethics approval from the Education and Health Sciences Research Ethics Committee 2023_06_09_EHS.
If you have any concerns about this study and wish to contact someone independent you may contact:
Chair Education and Health Sciences Research Ethics Committee
EHS Faculty Office
University of Limerick
Tel (061) 234101

○ I have read and understand the above introduction.

Personal Info Q1 Please enter your date of birth below in the dd/mm/yyyy format.

____

Personal Info Q2 Please enter the name of the school you attend below.

____

Personal Info Q3 Please select what school year you completed in May.

○ 4th Year

○ 5th Year

○ 6th Year

Personal Info Q4 What is your primary playing position?

- ○ 1 - Tight Head Prop
- ○ 2 - Hooker
- ○ 3 - Loose Head Prop
- ○ 4 - Second Row
- ○ 5 - Second ow
- ○ 6 - Blind Side Flanker
- ○ 7 - Open Side Flanker
- ○ 8 - Number 8
- ○ 9 - Scrum-Half
- ○ 10 - Fly-Half
- ○ 11 - Left Wing
- ○ 12 - Inside Centre
- ○ 13 - Outside Centre
- ○ 14 - Right Wing
- ○ 15 - Full-Back

End of Block: Introduction

Start of Block: Consent

Consent Form Read the following carefully and tick the I agree to patriciate before beginning the questionnaire.
· I declare that I agree to take part in research for the project entitled: Investigating nutrition and hydration knowledge and practice among age grade rugby union players.
· I declare that I have been fully briefed on the nature of this study and my role in it and have been given the opportunity to ask questions before agreeing to participate.
· The nature of my participation has been explained to me, and I have full knowledge of how the information collected will be used.
· I am aware that such information may also be used in research articles or in dissemination activities such as conference proceedings.
· I am aware that data sets generating from this research may potentially be shared with the wider researcher community in alignment with Open Science principles for sharing research findings and increasing their resource value.

· I fully understand that there is no obligation on me to participate in this study.
· I fully understand that I am free to withdraw my participation without having to explain or give a reason, up to the point that the questionnaire is completed and submitted.
· I understand that once I complete and submit the questionnaire, due to the anonymous nature of the questionnaire, I will no longer be able to withdraw my responses.
· I acknowledge that the researcher guarantees that they will not use my name or any other information, that would identify me in any outputs of the research.

○ I understand all the above and I agree to participate in this study.

○ I do not wish to participate in this study.

End of Block: Consent

Start of Block: Weight Management

Q1.1 Which nutrient do you think has the most energy (kilojoules/calories) per 100 grams (3.5 ounces)?

○ Carbohydrate

○ Protein

○ Fat

○ Not sure

Q1.2 Do you agree or disagree with the following statements about weight loss?

	Agree	Disagree	Not Sure
Having the lowest weight possible benefits endurance performance in the long term	○	○	○
Eating more protein is the most important dietary change if you want to have more muscle	○	○	○
Eating more energy from protein than you need can make you put on fat	○	○	○

Q1.3 Do you think the diet changes below are good ways to lose weight?

	Yes	No	Not Sure
Swapping carbohydrates/energy dense foods for low-energy foods like vegetables	○	○	○
Eating margarine instead of butter	○	○	○
Eating protein bars and shakes instead of yogurts, muesli/granola bars and fruits	○	○	○
Choosing lower glycemic index (GI) carbohydrates to help regulate appetite	○	○	○

Q1.4 If they want to lose weight, athletes should:

○ Eat less than 50 grams (1.7 ounces) of carbohydrate per day

○ Eat less than 20 grams (0.7 ounces) of fat per day

○ Eat less calories/kilojoules than your body needs

○ Not sure

Q1.5 To ensure they meet their energy (kilojoule/calorie) requirements, all athletes should:

○ Plan their diet based on their age, gender, body size, sport and training program

○ Eat based on their natural hunger and fullness signals

○ Eat at least 8000 kilojoules (2000 calories) per day

○ Eat more foods that have lots of carbohydrate

○ Not sure

Q1.6 Which is a better recovery meal option for an athlete who wants to put on muscle?

- A 'mass gainer' protein shake and 3 - 4 scrambled eggs
- Pasta with lean beef and vegetable sauce, plus a dessert of fruit, yoghurt and nuts
- A large piece of grilled chicken with a side salad (lettuce, cucumber, tomato)
- A large steak and fried eggs
- Not sure

Q1.7 Which is a better recovery meal option for an athlete who wants to lose weight?

- A side salad with no dressing (lettuce, cucumber, tomato)
- A pure whey protein isolate (WPI) shake made on water
- A mixed meal that includes a small-moderate serving of meat and carbohydrate (e.g. small bowl pasta with lean mincemeat and vegetable sauce) plus a large side salad
- Not sure

End of Block: Weight Management

Start of Block: Macronutrients

Q2.1 An athlete doing a moderate to high-intensity endurance training program for about two hours should eat…

- 1 - 3 g carbohydrate per kg (0.016 - 0.048 ounces per lb) body weight per day
- 5-7 g, increasing up to 10 g/kg with intense training/competition loads 15 - 25% of total daily kilojoule/calorie intake as carbohydrate
- 75 - 85% of total daily kilojoule/calorie intake as carbohydrate
- Not sure

Q2.2 Which options have enough carbohydrate for recovery from about 1 hour of high intensity aerobic exercise? Assume the athlete weighs about 70kg and has an important training session again tomorrow.

	Enough	Not enough	Not Sure
1 medium banana	○	○	○
1 cup cooked quinoa and 1 tin tuna	○	○	○
1 cup plain yoghurt	○	○	○
1 cup baked beans on two slices of bread	○	○	○

Q2.3 Which food has the most carbohydrate?

○ 1 cup (168 g/5.6 ounces) boiled rice

○ 2 slices of white sandwich loaf bread

○ 1 medium (150 g/ 5 ounces) boiled potato

○ 1 medium (150 g/5 ounces) ripe banana

○ Not sure

Q2.4 Do you agree or disagree with these statements about fat?

	Agree	Disagree	Not Sure
The body needs fat to fight off sickness	○	○	○
Athletes should not eat more than 20g of fat per day	○	○	○
When we increase the intensity of exercise, the % of fat we use as a fuel also increases	○	○	○
When we exercise at a low intensity, our body mostly uses fat as a fuel	○	○	○

Q2.5 Do you think these foods are high in fat?

	Yes	No	Not Sure
Cheddar cheese	○	○	○
Margarine	○	○	○
Mixed nuts	○	○	○
Honey	○	○	○

Q2.6 Do you agree or disagree with the statements about protein?

	Agree	Disagree	Not Sure
Protein is the main fuel that muscles use during exercise	○	○	○
Vegetarian athletes can meet their protein requirements without the use of protein supplements	○	○	○
An experienced athlete needs more protein than a young athlete who is just starting training	○	○	○
The body has a limited ability to use protein for muscle protein synthesis	○	○	○
A balanced diet with enough kilojoules/calories (energy) has enough protein for most athletes	○	○	○

Q2.7 Which food has the most protein?

○ 2 eggs

○ 100g (3 ounces) raw skinless chicken breast

○ 30g (1 ounce) almonds

○ Not sure

Q2.8 The protein needs of a 100 kg (220 lb) well trained resistance athlete are closest to:

- 100g (1g/kg)
- 150g (1.5g/kg)
- 500g (5g/kg)
- They should eat as much protein as possible
- Not sure

Q2.9 Which of these foods do you think have enough protein to promote muscle growth after a bout of resistance exercise?

	Enough	Not enough	Not Sure
100g (3 ounces) chicken breast	○	○	○
300g (1 ounce) Yellow cheese	○	○	○
1 cup baked beans	○	○	○
1/2 cup cooked quinoa	○	○	○

Q2.10 Do you think these foods have all the essential amino acids needed by the body?

	Yes	No	Not Sure
Beef steak	○	○	○
Eggs	○	○	○
Lentils	○	○	○
Cow's milk	○	○	○

Q2.11 The amount of protein in skim milk compared to full cream milk is:

- ○ Much less
- ○ About the same
- ○ Much more
- ○ Not sure

End of Block: Macronutrients

Start of Block: Micronutrients

Q3.1 Do you agree or disagree with these statements on vitamins and minerals?

	Agree	Disagree	Not Sure
Calcium is the main component of bone	○	○	○
Vitamin C is an anti-oxidant	○	○	○
Thiamine (Vitamin B1) is needed to take oxygen to muscles	○	○	○
Iron is needed to turn food into usable energy	○	○	○
Vitamin D enhances calcium absorption	○	○	○
Meat, chicken and fish are good sources of zinc	○	○	○
Wholegrain foods are good sources of vitamin C	○	○	○
Fruit and vegetables are good sources of calcium	○	○	○
Fatty fish is a good source of vitamin D	○	○	○
Women who have a monthly period need more iron than men	○	○	○
Athletes aged 15 to 24 years need 500 mg of calcium each day	○	○	○
A fit person eating a balanced diet can improve their athletic performance by eating more vitamins and minerals from food	○	○	○
Vitamins contain energy (kilojoules/calories)	○	○	○

End of Block: Micronutrients

Start of Block: Sports Nutrition

Q4.1 Athletes should drink water to:

- ○ Keep plasma (blood) volume stable
- ○ Stop dry mouth
- ○ Allow proper sweating
- ○ All of the above
- ○ Not sure

Q4.2 Experts think that athletes should:

- ○ Drink 50 - 100 ml (1.7 - 3.3 fluid ounces) every 15 - 20 minutes
- ○ Suck on ice cubes rather than drinking during training
- ○ Drink sports drinks (e.g. Powerade) rather than water when exercising
- ○ Drink to a plan, based on body weight changes during training sessions performed in a similar climate
- ○ Not sure

Q4.3 How much sodium (salt) should fluid consumed for hydration purposes (during exercise) contain?

- ○ At least 11 - 25 mmol/L (~ 250 - 575 mg/L)
- ○ At least 4 - 8 mmol/L (~ 90 - 185 mg/L)
- ○ None
- ○ Not sure

Q4.4 Before competition, athletes should eat foods that are high in:

- ○ Fluids, fat and carbohydrate
- ○ Fluids, fibre and carbohydrate
- ○ Fluids and carbohydrate
- ○ Not sure

Q4.5 Do you agree or disagree with the statements on carbohydrate?

	Agree	Disagree	Not Sure
Eating carbohydrates when you exercise makes it harder to build strength and muscles	○	○	○
In events lasting 60 - 90 minutes, 30- 60 g (1.0 - 2.0 ounces) of carbohydrates should be eaten per hour	○	○	○
Eating carbohydrates when you exercise will help keep blood sugar levels stable	○	○	○

Q4.6 Some athletes get a sore stomach if they eat during exercise. What might make stomach pain worse?

○ Having energy gels rather than water or sports drinks

○ Having small amounts of water at a time

○ Having sports drinks with different types of carbohydrates (e.g. fructose and sucrose)

○ Not sure

Q4.7 During a competition, athletes should eat foods that are high in:

○ Fluids, fibre and fat

○ Fluids and protein

○ Fluids and carbohydrate

○ Not sure

Q4.8 Which is the best snack to have during an intense 90-minute training session?

○ A protein shake

○ A ripe banana

○ 2 boiled eggs

○ A handful of nuts

○ Not sure

Q4.9 After a competition, athletes should eat foods that are high in?

- Protein, carbohydrate and fat
- Only protein
- Only carbohydrate
- Carbohydrate and protein
- Not sure

Q4.10 How much protein do you think experts say athletes should eat after resistance exercise?

- 0.3 g/kg body weight (~ 15 - 25 g [0.53 - 0.88 ounces] for most athletes)
- 1.0 g/kg body weight (~ 50 - 100 g [1.9 - 2.3 ounces] for most athletes)
- 1.5 g/kg body weight (~ 150 – 130 g [5.3 – 10.6 ounces] for most athletes)
- Not sure

End of Block: Sports Nutrition

Start of Block: Supplementation

Q5.1 Do you agree or disagree with the statements about vitamin and mineral supplements?

	Agree	Disagree	Not Sure
Vitamin C should always be taken by athletes	○	○	○
B vitamins should be taken if energy levels are low	○	○	○
Salt tablets should be taken by athletes that get cramps when they exercise	○	○	○
Iron tablets should be taken by all athletes who feel tired and are pale	○	○	○

Q5.2 All supplements are tested to make sure they are safe, don't have any contamination.

- Agree
- Disagree
- Not sure

Q5.3 Supplement labels may sometimes say things that are not true.

- Agree
- Disagree
- Not sure

Q5.4 Do you agree or disagree with the statements about supplements?

	Agree	Disagree	Not Sure
Creatine makes the brain think that exercise feels easier	○	○	○
Caffeine makes muscles able to work harder even without more oxygen	○	○	○
Beetroot juice (nitrates) makes muscles feel less sore after exercise	○	○	○
Beta-Alanine can decrease how much acid muscles make during intense exercise	○	○	○

Q5.5 Which supplement does not have enough evidence in relation to improving body composition or sporting performance?

- Caffeine
- Ferulic acid
- Bicarbonate
- Leucine
- Not sure

Q5.6 WORLD ANTI-DOPING AGENCY (WADA) bans the use of….

- Caffeine
- Bicarbonate
- Carnitine
- Testosterone
- Not sure

End of Block: Supplementation

Start of Block: Alcohol

Q6.1 How much ethanol (pure alcohol) is there in a standard drink?

- 1 - 2 g / 0.03 - 0.06 fluid ounces
- 8 - 14 g / 0.3 - 0.6 fluid ounces
- 30 - 50 g / 1.2 - 2.0 fluid ounces
- Not sure

Q6.2 Which is an example of a "Standard Drink"?

- 30 - 45 ml / 1 - 1.5 fluid ounces of pure spirits
- One quarter of a bottle (175 ml / 6 fluid ounces) of red wine
- A pint (425 ml / 14 fluid ounces) of full strength beer
- Not sure

Q6.3 Do you think alcohol can make you put on weight?

- Yes
- No
- Not sure

Q6.4 How many drinks do you think experts say are the most we should have in one day?

- ○ Two
- ○ Three
- ○ Four
- ○ Not sure

Q6.5 Do you agree or disagree with the statements on alcohol?

	Agree	Disagree	Not Sure
If someone does not drink at all during the week, it is okay for them to have five or more drinks on a Friday or Saturday night	○	○	○
Drinking lots of alcohol can make it harder to recover from injury	○	○	○
Alcohol makes you urinate more	○	○	○

Q6.6 "Binge drinking" (also referred to as heavy episodic drinking) is defined as:

- ○ Having two or more standard alcoholic drinks on the same occasion
- ○ Having four to five or more standard alcoholic drinks on the same occasion
- ○ Having seven to eight or more standard alcoholic drinks on the same occasion
- ○ Not sure

End of Block: Alcohol
