# Supplementary material for: Investigating Nutrition and Hydration Knowledge and Practice among a Cohort of Age-Grade Rugby Union Players
Source: Nutrients. 2024 Feb 14;16(4):533. doi: 10.3390/nu16040533 (PMC10891571; doi:10.3390/nu16040533)
Supplement: Supplementary file 1 [file nutrients-16-00533-s001.zip › File S2. Copy of The Hydration Assessment Questionnaire.pdf]

File S2. Copy of The Hydration Assessment Questionnaire

Start of Block: Introduction

Thank you for participating in this study entitled: Investigating Nutrition and Hydration Knowledge and Practice Among Age Grade Rugby Union Players. This questionnaire examines your hydration knowledge and practices. It should take 15 to 20 minutes to complete.

Our aim for this research is to investigate nutrition and hydration knowledge and practice among age grade rugby union players participating in the Munster Rugby Age-Grade Player Development Programme. Associations with habitual hydration status and / or dietary practices (energy and macronutrient intakes) and sports performance (speed, strength, endurance) will be investigated.

Please read each question carefully and answer each question as truthfully as possible. If you do not know the answer to a particular question do not guess. Use the 'Not Sure' option for questions you do not know the answer to.

If you wish to ask any more questions about the research process you can contact the researchers at the following: Catherine Norton - catherine.norton@ul.ie or Ronan Mangan - 22290974@studentmail.ul.ie.

This research study has received Ethics approval from the Education and Health Sciences Research Ethics Committee 2023_06_09_EHS.
If you have any concerns about this study and wish to contact someone independent you may contact:
Chair Education and Health Sciences Research Ethics Committee
EHS Faculty Office
University of Limerick
Tel (061) 234101

Consent Form Read the following carefully and tick the I agree to patriciate before beginning the questionnaire.
· I declare that I agree to take part in research for the project entitled: Investigating nutrition and hydration knowledge and practice among age grade rugby union players.
· I declare that I have been fully briefed on the nature of this study and my role in it and have been given the opportunity to ask questions before agreeing to participate.
· The nature of my participation has been explained to me, and I have full knowledge of how the information collected will be used.
· I am aware that such information may also be used in research articles or in dissemination activities such as conference proceedings.
· I am aware that data sets generating from this research may potentially be shared with the wider researcher community in alignment with Open Science principles for sharing research findings and increasing their resource value.
· I fully understand that there is no obligation on me to participate in this study.
· I fully understand that I am free to withdraw my participation without having to explain or give a reason, up to the point that the questionnaire is completed and submitted.
· I understand that once I complete and submit the questionnaire, due to the anonymous nature of the questionnaire, I will no longer be able to withdraw my responses.
· I acknowledge that the researcher guarantees that they will not use my name or any other information, that would identify me in any outputs of the research.

○ I understand all the above and I agree to participate in this study.

○ I do not wish to participate in this study.

Personal Info Q1 Please enter your date of birth below in the dd/mm/yyyy format.

____

Personal Info Q2 Please enter the name of the school you attend below.

____

Personal Info Q3 Please select what school year you are currently in.

- ○ 4th Year
- ○ 5th Year
- ○ 6th Year

Personal Info Q4 What position do you play in rugby?

- ○ 1 Tight head prop
- ○ 2 Hooker
- ○ 3 Loose head prop
- ○ 4 Second row
- ○ 5 Second row
- ○ 6 Blink side flanker
- ○ 7 Open side flanker
- ○ 8 Number 8
- ○ 9 Scrum-half
- ○ 10 Fly-half
- ○ 11 Left wing
- ○ 12 Inside center
- ○ 13 Outside center
- ○ 14 Right wing
- ○ 15 Full-back

Q5 Please check the correct box that applies to you?

- ○ I have not had any hydration/fluid education within sports.
- ○ I have a good level of knowledge in relation to hydration/fluid within sports.
- ○ Please write in the text box below (YES/NO) if you have received or completed educational classes based on hydration/fluid within sports. ____

End of Block: Introduction

Start of Block: Knowledge

Knowledge PLEASE ANSWER THE FOLLOWING QUESTIONS BY PICKING ONE ANSWER EITHER TRUE OR FALSE

Q1 Using salt tablets keeps athletes from getting dehydrated during training and competition.

- ○ True
- ○ False

Q2 Thirst is a strong indicator of dehydration.

- ○ True
- ○ False

Q3 Dehydration decreases athletic performance.

- ○ True
- ○ False

Q4 An athlete should not drink any water or fluids during training.

- ○ True
- ○ False

Q5 Coaches should not let players drink any water or fluids during training.

- ○ True
- ○ False

Q6 Coaches should not let players drink any water or fluids during competition/matches.

- ○ True
- ○ False

Q7 It is important for fluids to be readily available to athletes during training.

- ○ True
- ○ False

Q8 It is important for fluids to be readily available to athletes during competition/matches.

- ○ True
- ○ False

Q9 Within 2 hours after exercise, athletes should drink a sports drink.

- ◯ True
- ◯ False

Q10 Sports drinks are better than water because they restore glycogen in muscles.

- ◯ True
- ◯ False

Q11 An athlete should drink 500 – 600 ml of water or a sports drink a couple of hours before competition/matches.

- ◯ True
- ◯ False

Q12 An athlete should drink 200 – 300 ml of water or a sports drink 10-20 min before competition/matches.

- ◯ True
- ◯ False

Q13 When exercising for more than 1 hour, an athlete should drink a sports drink rather than water.

- ◯ True
- ◯ False

Q14 By monitoring the colour of urine, an athlete can judge if he/she is dehydrated

- ◯ True
- ◯ False

Q15 A good way for an athlete to determine how much water or sports drink to consume after training is to obtain body weight before and after training.

- ◯ True
- ◯ False

Q16 Excessive sweating, thirst, and muscle cramping are signs of dehydration.

- ◯ True
- ◯ False

Q17 More than two drinks of alcohol the day before training and/or competition/matches can lead to dehydration.

- True
- False

End of Block: Knowledge

Start of Block: Attitude

Attitudes PLEASE ANSWER THE FOLLOWING QUESTIONS BY PICKING ONE ANSWER OF THE OPTIONS BELOW

- Strongly Disagree
- Disagree
- Undecided
- Agree
- Strongly Agree

Q1 I believe using salt tablets keeps me from getting dehydrated during training and competition.

- Strongly Disagree
- Disagree
- Undecided
- Agree
- Strongly Agree

Q2 I believe dehydration decreases my athletic performance.

- Strongly Disagree
- Disagree
- Undecided
- Agree
- Strongly Agree

Q3 I believe I can rely on thirst alone as an indicator of dehydration.

- Strongly Disagree
- Disagree
- Undecided
- Agree
- Strongly Agree

Q4 I believe no water or fluids should be consumed during training.

- Strongly Disagree
- Disagree
- Undecided
- Agree
- Strongly Agree

Q5 I believe my coach should not let athletes drink any fluids during training.

- Strongly Disagree
- Disagree
- Undecided
- Agree
- Strongly Agree

Q6 I believe my coach should not let athletes drink any fluids during competition/matches.

- Strongly Disagree
- Disagree
- Undecided
- Agree
- Strongly Agree

Q7 I believe fluids should be readily available to me during training.

- Strongly Disagree
- Disagree
- Undecided
- Agree
- Strongly Agree

Q8 I believe fluids should be readily available to me during competition/matches.

- Strongly Disagree
- Disagree
- Undecided
- Agree
- Strongly Agree

Q9 I believe I should drink a sports drink within 2 hours after exercise.

- Strongly Disagree
- Disagree
- Undecided
- Agree
- Strongly Agree

Q10 I think sports drinks are better than water because they restore glycogen in muscles.

- Strongly Disagree
- Disagree
- Undecided
- Agree
- Strongly Agree

Q11 I believe I should drink 500 – 600 ml of water or a sports drink a couple of hours before competition/matches.

- ○ Strongly Disagree
- ○ Disagree
- ○ Undecided
- ○ Agree
- ○ Strongly Agree

Q12 I believe I should drink 200 – 300 ml of water or a sports drink 10-20 minutes before competition/matches.

- ○ Strongly Disagree
- ○ Disagree
- ○ Undecided
- ○ Agree
- ○ Strongly Agree

Q13 I believe when exercising for more than 1 hour, I should drink a sports drink rather than water.

- ○ Strongly Disagree
- ○ Disagree
- ○ Undecided
- ○ Agree
- ○ Strongly Agree

Q14 I believe by monitoring the colour of my urine, I can judge if I am dehydrated.

- ○ Strongly Disagree
- ○ Disagree
- ○ Undecided
- ○ Agree
- ○ Strongly Agree

Q15 I believe weighing myself before and after training is a good way to determine how much fluid I lost.

- Strongly Disagree
- Disagree
- Undecided
- Agree
- Strongly Agree

Q16 I believe excessive sweating, thirst, and muscle cramping are signs of dehydration.

- Strongly Disagree
- Disagree
- Undecided
- Agree
- Strongly Agree

Q17 I believe drinking more than two drinks of alcohol the day before competing can lead to dehydration.

- Strongly Disagree
- Disagree
- Undecided
- Agree
- Strongly Agree

End of Block: Attitude

Start of Block: Behavior

Behavior PLEASE ANSWER THE FOLLOWING QUESTIONS BY PICKING ONE ANSWER EITHER TRUE OR FALSE

Q1 I ingest salt tablets which keeps me from getting dehydrated during training and competition.

- Yes
- No

Q2 I drink plenty of fluids so my athletic performance will not decrease due to dehydration.

- Yes
- No

Q3 I use thirst alone as a way to tell if I am dehydrated.

- Yes
- No

Q4 I do not drink water or some type of fluid during training.

- Yes
- No

Q5 My coach does not allow me to drink fluids during training.

- Yes
- No

Q6 My coach does not allow me to drink fluids during competition/matches.

- Yes
- No

Q7 Fluids are readily available to me during training.

- Yes
- No

Q8 Fluids are readily available to me during competition/matches.

- Yes
- No

Q9 Within 2 hours after exercise, I drink a sports drink.

- Yes
- No

Q10 I drink sports drinks rather than water to restore glycogen in my muscles.

- Yes
- No

Q11 I drink 500 – 600 ml of water or a sports drink a couple of hours before competition/matches.

- Yes
- No

Q12 I drink 200 – 300 ml of water or a sports drink 10-20 minutes before competition/matches.

- Yes
- No

Q13 I drink sports drinks rather than water when exercising for more than an hour.

- Yes
- No

Q14 I use the colour of my urine to determine if I am dehydrated.

- Yes
- No

Q15 I weigh myself before and after training to see how much weight I have lost from sweating and use this to determine how much water or sports drink to consume.

- Yes
- No

Q16 I use excessive sweating, thirst, and muscle cramping to warn me if I am becoming dehydrated.

- Yes
- No

Q17 I drink more than 2 drinks of alcohol the day before competition/matches.

- Yes
- No

End of Block: Behavior
