# Supplementary material for: Investigating Nutrition and Hydration Knowledge and Practice among a Cohort of Age-Grade Rugby Union Players
Source: Nutrients. 2024 Feb 14;16(4):533. doi: 10.3390/nu16040533 (PMC10891571; doi:10.3390/nu16040533)

File S3. Copy of Study Background and Libro App Information Presentation

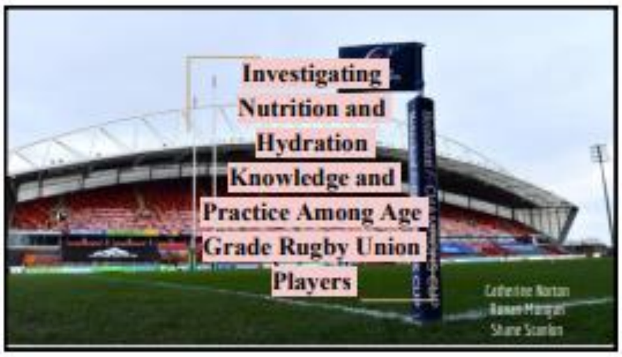

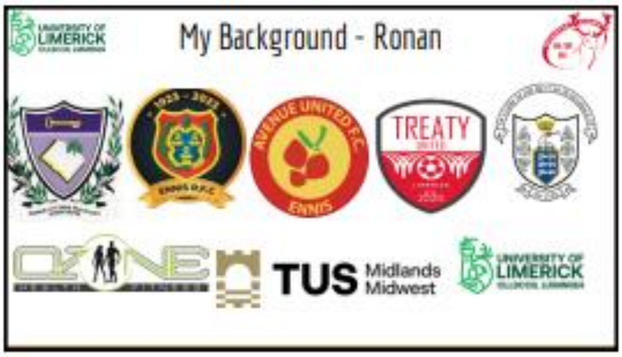

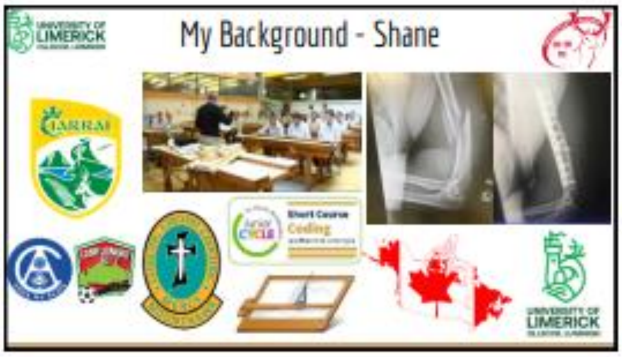

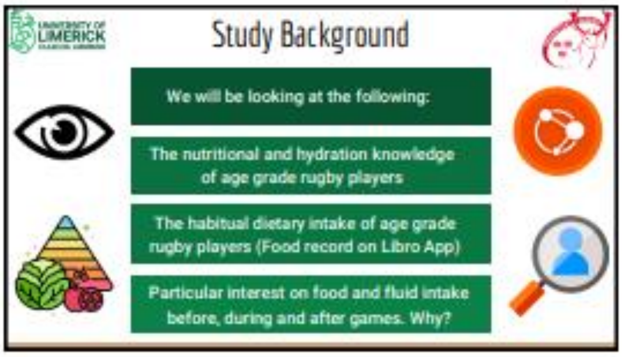

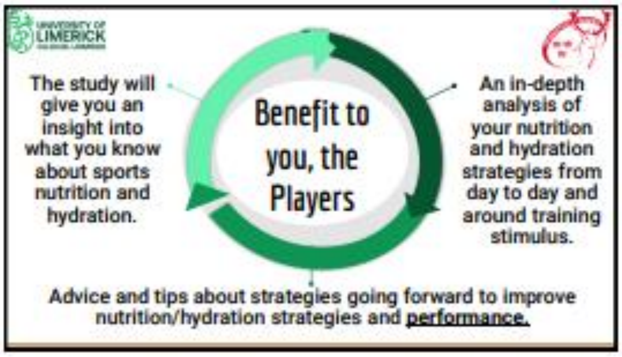

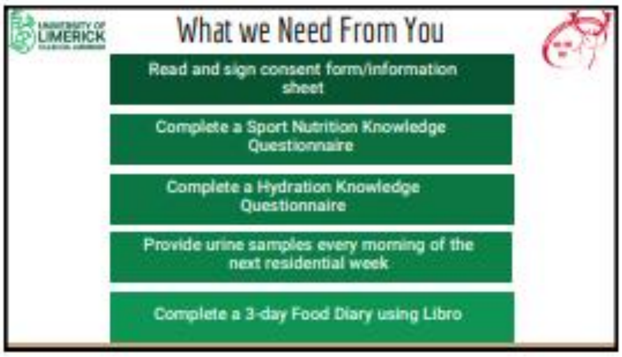

Questionnaires

- Two short *separate* questionnaires.
- Should take 15/20 mins max each.
- Can be completed using your phones.
- Links to the questionnaires will be provided.
- If you don't know any answers don't guess, use the *Not Sure* option.
- The more honest you are the better.

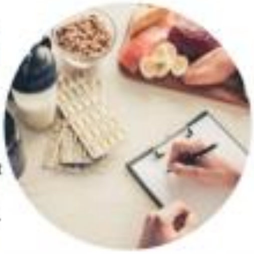

Urine Samples

- Samples taken every morning.
- You will be given a cup with the same number every day.
- Hydration status will be recorded using a refractometer.
- Important not to change you hydration strategies just because you are being tested.
- Stick to your usual routine.

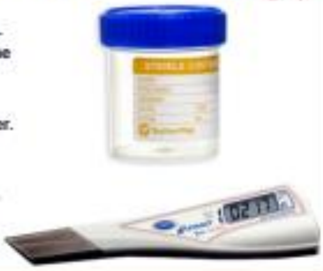

Food Diary - Libro App

- 3-day food diary using the Libro App.
- Log <u>everything</u> you consume each day, <u>food and fluid.</u>
- Important not to change your dietary intake over the 3-day period.
- At least one rest day and one training/match day needs to be logged.
- <u>On training/match days it is important to log your pre and post meals.</u>
- Stick to your usual routine.

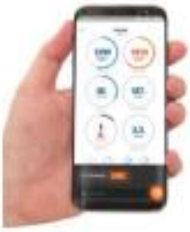

Libro App

- I will send an email invite to everyone. I will be using the email address you provided Munster with at the start of the program.
- You will receive the email on the right prompting you to download the Libro App.

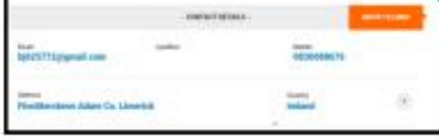

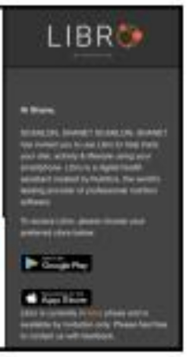

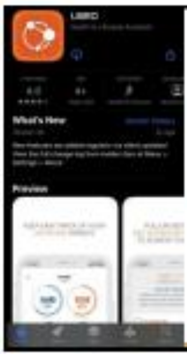

Libro App

- The image on the left is the Libro App in the App Store. Download the app.
- The image on the right is the login screen which will appear the first time you open the app.

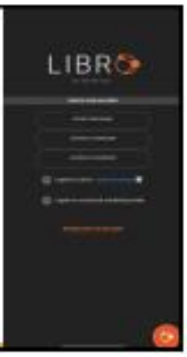

Libro App

- Create an account with the same email address you provided Munster with.
- Choose a username.
- Create a password. I would suggest *Password123* for everyone.
- Click the tick to agree to the terms of service.
- Once you have all the above done a white tick will appear in the orange circle on the bottom right of the screen.
- Press the tick and you will be logged into the app.

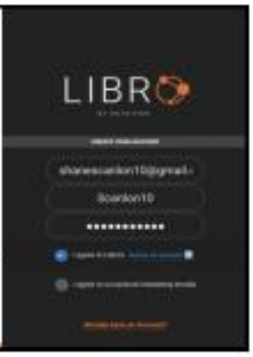

Supplement: Supplementary file 1 [file nutrients-16-00533-s001.zip › File S3. Copy of Study Background and Libro App Information Presentation.pdf]
