# Supplementary material for: Investigating Nutrition and Hydration Knowledge and Practice among a Cohort of Age-Grade Rugby Union Players
Source: Nutrients. 2024 Feb 14;16(4):533. doi: 10.3390/nu16040533 (PMC10891571; doi:10.3390/nu16040533)
Supplement: Supplementary file 1 [file nutrients-16-00533-s001.zip › Table S1. Participants Training Schedule and Data Collection Process.pdf]

Table S1. Participants Training Schedule and Data Collection Process

Week One Residential Training Schedule at the University of Limerick and Data Collection Process				
**Time**	**Monday**	**Tuesday**	**Thursday**	**Friday**
08:00		Arrive & Markers		Arrive & Markers
08:30		***Urine Sample 2***		***Urine Sample 4***
09:00		Forwards Gym/Backs Pitch Speed Session		Team Meeting & Strapping
09:30	Arrive, Markers & ***Urine Sample 1***	Backs Gym/Forwards Pitch Speed Session		Rugby Pitch Session – 60mins
10:00	Anthropometric Data Collection	Snacks BYO & Strapping	Arrive & Markers	
10:30	Team Meeting & Strapping	Rugby Pitch Session – 60mins	***Urine Sample 3***	Snacks BYO
11:00	Rugby Pitch Session – 75mins		Team Meeting & Strapping	Backs Gym/Forwards Pitch Speed Session
11:30				
12:00		Debrief	Rugby Challenge Match – 90mins	Forwards Gym/Backs Pitch Speed Session
12:30	Lunch	Take Away Lunch & Depart		
13:00	Backs Gym/Forwards Speed Session			Debrief
13:30				Take Away Lunch & Depart
14:00	Forwards Gym/Backs Speed Session		Lunch	
14:30				
15:00	Accommodation		Accommodation	
P.M.	Education – 60mins	***Distribution of HAQ***	Education – 60mins	
**Week Two Non-Residential Training Schedule at the University of Limerick and Musgrave Park, Cork and Data Collection Process**				
**Time**	**Monday**	**Tuesday**	**Thursday**	**Friday**
09:00			Arrive & Markers	
09:30			Team Meeting Review	Arrive & Markers
10:00	Arrive & Markers	Arrive & Markers	Strapping	Forwards Gym/Backs Pitch Speed Session
10:30	Team Meeting & Strapping	***Urine Sample 5***	Rugby Pitch Session – 60mins	Backs Gym/Forwards Pitch Speed Session
11:00	Rugby Pitch Session – 75mins	Team Meeting & Strapping		Snacks BYO & Strapping
11:30		Rugby Challenge Match – 90mins	Snacks BYO	Rugby Pitch Session – 60mins
12:00			Backs Gym/Forwards Pitch Speed Session	
12:30	Snacks BYO & Depart		Forwards Gym/Backs Pitch Speed Session	Debrief
13:00		Debrief	Debrief	Take Away Lunch & Depart
13:30		Take Away Lunch & Depart	Take Away Lunch & Depart	
14:00				
P.M.	***Distribution of NSKQ***	Distribution of Food Record Instructions<br>***Wednesday –Food Record Provisional Day 1***	***Food Record Provisional Day 1 or 2***	***Food Record Provisional Day 2 or 3***

*BYO bring your own, Monday and Thursday training during week two took place in Musgrave Park, Cork, with Tuesday and Friday training taking place in the University of Limerick, Food Records began on Wednesday of week two which was a rest day, Data collection points in **Bold and Italics**, participants began food records on Wednesday or Thursday of week two*
